# Tumor NLRP3-Derived IL-1β Drives the IL-6/STAT3 Axis Resulting in Sustained MDSC-Mediated Immunosuppression

**DOI:** 10.3389/fimmu.2021.661323

**Published:** 2021-08-31

**Authors:** Isak W. Tengesdal, Alberto Dinarello, Nicholas E. Powers, Matthew A. Burchill, Leo A. B. Joosten, Carlo Marchetti, Charles A. Dinarello

**Affiliations:** ^1^Department of Medicine, University of Colorado Denver, Aurora, CO, United States; ^2^Department of Internal Medicine and Radboud Institute of Molecular Life Sciences (RIMLS), Radboud University Medical Center, Nijmegen, Netherlands; ^3^Dipartimento di Biologia, Università degli Studi di Padova, Padova, Italy

**Keywords:** interleukin-1β, NLRP3, interleukin-6, signal transducer and activator of transcription 3, myeloid-derived suppressor cells

## Abstract

Tumors evade the immune system by inducing inflammation. In melanoma, tumor-derived IL-1β drives inflammation and the expansion of highly immunosuppressive myeloid-derived suppressor cells (MDSCs). Similar in many tumors, melanoma is also linked to the downstream IL‐6/STAT3 axis. In this study, we observed that both recombinant and tumor-derived IL-1β specifically induce pSTAT3(Y705), creating a tumor-autoinflammatory loop, which amplifies IL-6 signaling in the human melanoma cell line 1205Lu. To disrupt IL-1β/IL-6/STAT3 axis, we suppressed IL-1β-mediated inflammation by inhibiting the NOD-like receptor protein 3 (NLRP3) using OLT1177, a safe-in-humans specific NLRP3 oral inhibitor. *In vivo*, using B16F10 melanoma, OLT1177 effectively reduced tumor progression (*p*< 0.01); in primary tumors, OLT1177 decreased pSTAT3(Y705) by 82% (*p*<0.01) and *II6* expression by 53% (*p*<0.05). Disruption of tumor-derived NLRP3, either pharmacologically or genetically, reduced STAT3 signaling in bone marrow cells. In PMN-MDSCs isolated from tumor-bearing mice treated with OLT1177, we observed significant reductions in immunosuppressive genes such as *Pdcd1l1*, *Arg1*, *Il10* and *Tgfb1*. In conclusion, the data presented here show that the inhibition of NLRP3 reduces IL-1β induction of pSTAT3(Y705) preventing expression of immunosuppressive genes as well as activity in PMN-MDSCs.

## Introduction

An evolving understanding of malignant tumor progression reveals that tumors evade the immune system through several mechanisms including NK and T cell exhaustion (1, 2), regulatory T cell induction (3, 4), expression of inhibitory receptors (5), tumor-associated macrophage alterations (6) and recruitment of myeloid-derived suppressor cells (MDSCs) (7). Specifically, MDSCs facilitate tumor immune evasion by inhibiting anti-tumor responses of both T and NK cells as well as induction of regulatory T cells (8–10). MDSC expansion is mediated through chronic tumor-associated inflammation (for example, pro-inflammatory cytokines). MDSCs also suppress T cell anti-tumor actions *via* programmed death-ligand 1 (PD-L1), arginase (Arg-1), interleukin-10 (IL-10) and transforming growth factor beta (TGF-β) (11, 12). In melanoma, patients with advanced stage melanoma have elevated circulating levels of the proinflammatory cytokines IL‐1β and IL‐6, which correlate with poor prognosis (13, 14). Moreover, inflammatory cytokines such as IL‐1β and IL‐6 have been associated with MDSCs expansion and activity (15, 16). Nevertheless, the molecular mechanisms linking inflammation to MDSCs expansion and immunosuppressive gene regulation remain unclear.

In advanced stage cancers, IL-6 is highly associated with disease progression (17–19). Canonically, IL‐6 binds to either the membrane bound IL-6 receptor-α chain or soluble IL-6. Subsequently, these complexes bind ADAM10 or ADAM17 resulting in JAK/STAT activation and gene transcription. STAT3 is a transcription factor that regulates several unrelated biological processes such as maintenance of stem cell pluripotency, regeneration, autophagy, cell proliferation, and wound healing (18). In several cancers including melanoma, STAT3 is a prognostic marker predicting poor outcomes (20–22). STAT3 functions take place with the phosphorylation of tyrosine 705 (Y705) (23). Once phosphorylated, STAT3 dimerizes and enters the nucleus. There dimerized pSTAT3 binds specific DNA elements or binds with other transcription factors, modulating their activity (termed tethering) (24). Importantly, the trigger for Y705 phosphorylation is linked to IL‐6 and Janus kinases.

Many tumors exhibit elevated levels of IL-6, which in turn activate the JAK/STAT3 signaling cascade in infiltrating immune cells, resulting in immunosuppressive activity (25–27). Moreover, aberrant IL-6 production is strongly associated with the expansion and activation of MDSCs (15, 19, 28). Additionally, immunosuppressive activity of MDSCs, such as IL-10 release, PD-L1 and arginase expression are tightly associated with STAT3 phosphorylation linking the IL-6/STAT3 axis to gene expression in MDSCs (29–32). However, in the context of melanoma this cascade remains unknown.

Recently, we have reported constitutive NOD-like receptor protein 3 (NLRP3) activation and NLRP3 inflammasome formation in melanoma (16). NLRP3 is an intracellular pattern recognition receptor; upon NLRP3 activation, the inactive IL‐1β precursor is processed by caspase‐1 to active, mature IL‐1β (33). Cytokines associated with MDSC expansion such as IL-6 are induced by IL-1β (34–36). In this study, we investigate whether NLRP3-dependent IL-1β production observed in melanoma cells drives IL-6/STAT3 signaling and whether this influences MDSC activity.

## Methods

### Cell Culture

1205Lu and A357 human melanoma cells were cultured in RPMI, supplemented with 10% FBS, 100 units/ml penicillin, 0.1 mg/ml streptomycin. Cells were maintained in a humidified 5% CO_2_ atmosphere at 37°C. Cells were detached from flasks using 0.25% Trypsin, 0.1% EDTA (Corning, Manassas, VA), centrifuged and resuspended in complete media. The cells were plated at 2.5x10^5^ per well in a 24-wells plate and allowed to adhere overnight. The following day, the media were replaced with fresh RPMI, 10%FBS in presence and absence of the NLRP3 inhibitor OLT1177 (10 µM) (37). Supernatants were collected after 48 hours. In a separate set of experiments, IL-1β was added to the culture for stimulation (20 ng/ml; R&D Systems, Minneapolis, MN). Supernatants were collected after 24 hours. The murine melanoma cells B16F10 were cultured in DMEM GlutaMax, supplemented with 10%FBS, 100 units/ml penicillin, 0.1 mg/ml streptomycin. B16F10 NLRP3 knockout cells (B16F10 *nlrp3^-/-^*) (Synthego, Redwood City, CA) were genetically edited by CRISPR-Cas9 technology using the following guide RNA sequence: UUCCUCUAUGGUAUGCCAGG. 48 hours post-transfection editing efficiency was determined at 96% compared to the control samples using the following PCR and sequencing primers: F:TTTCCTGCCTCCATCTCCCA and R:TTCAGTGAAGGCGGGTTTCC.

### Cytokine Measurements

Cytokines were measured by specific DuoSet ELISAs according to the manufacturer’s instructions (R&D Systems).

### Western Blotting

1205Lu or A357 cells were cultured as previously described. Primary tumor and bone marrow cells were collected from tumor-tumor bearing. All cells were lysed in RIPA buffer (Sigma, St. Louis, MS, USA) supplemented with protease inhibitors (Roche, Indianapolis, IN), centrifuged at 13,000*g* for 20 min at 4°C and the supernatants were obtained. Protein concentration was determined in the clarified supernatant using Bio-Rad protein assay (Bio-Rad Laboratories, Hercules, CA). Proteins were electrophoresed on Mini-PROTEAN TGX 4−20% gels (Bio-Rad Laboratories) and transferred to nitrocellulose 0.2 μm (GE Water & Process Technologies, Feasterville-Trevose, PA). Membranes were blocked in 5% rehydrated non-fat milk in PBS-Tween 0.5% for 1 hour at room temperature. Primary antibodies for STAT3 and pSTAT3(Y705) (CellSignaling, Danvers, MA) were used in combination with peroxidase-conjugated secondary antibodies. A primary antibody against β-actin (Santa Cruz Biotechnology, Dallas, TX) was used to assess protein loading.

### *In Vivo* Model

Animal protocols were approved by the University of Colorado Animal Care and Use Committee. Wild type C57/Black 6 mice were purchased from The Jackson Laboratory (Bar Harbor, ME, USA). B16F10 (2 x10^5^) and B16F10 *nlrp3^-/-^* (2 x10^5^) cells were mixed with Matrigel (Corning) and then implanted subcutaneously (s.c.) in the hind quarter of mice. Mice were sacrificed 15 days after the plug instillation for molecular and cellular analysis. Tumor growth was recorded every three days. On day5 mice were treated daily by gavage of the NLRP3 specific inhibitor OLT1177 (generic dapansutrile, Olatec Therapeutics, LLC, New York, NY) at a concentration of 600mg/kg or the JAK inhibitor AZD1480 (Selleckchem, Houston, TX) (50mg/kg), starting on day 5 after melanoma cell implantation. Alternatively, OLT1177-enriched diet was used where denoted. Tumor volume was calculated using the formula V =1/2(LxWxH) where V is tumor volume in cubic millimeters (mm^3^). Dimensions were measured by electronic caliper on restrained mice. Tumors volumes were determined without knowledge of the experimental groups. Primary tumors and bone marrow were then assessed *via* western blotting or gene expression as described above.

### MDSC Isolation

PMN-MDSCs were isolated from bone and spleen of tumor-bearing described above and isolated using flow cytometry. Briefly, single-cell suspensions of bone marrow and spleens cells harvested and strained through 40 micron filters and were stained using anti-CD45 Pe/Cy7 (BioLegend, San Diego, CA), anti-CD11b BV785 (BioLegend), anti-Ly6G PacBlue (BioLegend), anti-Ly6C PerCp/Cy5.5 (BioLegend).

### T Cell MDSC Co-Culture

PMN-MDSCs were isolated from spleen of tumor-bearing mice described above using magnetic columns specific for MDSC isolation (Miltenyi Biotec, Germany). Naïve CD8+ T cells were isolated from spleen of non-tumor bearing mice using Mojosort kit (BioLegend) per manufacturer’s instructions. Isolated CD8+ T cells were then stained with VPD solution. 50,000 T cells were then co-cultured with 50,000 MDSCs and in 96-well plates coated with 5ug/mL anti-CD3/28 and incubated for 3 days. On day 3, supernatants were removed and assessed for cytokines and T cell proliferation was determined by flow cytometry.

### Gene Expression

Primary tumors or PMN-MDSCs (flow cytometry) were collected as described above. RNA was then isolated using Trizol (Thermo Fisher Scientific, Waltham, MA) and synthesized in cDNA using SuperScript III First-Strand (Thermo Fisher Scientific). Quantitative PCR (qPCR) was performed on cDNA using Power SYBR Green PCR master mix (Thermo Fisher Scientific) on Biorad CFX96 Real time system. Gene expression was assessed for the following mRNAs: *Socs3* (forward 5’-ATTTCGCTTCGGGACTAG-3’ and reverse 5’-AACTTGCTGTGGGTGACCAT-3’), *Klf4* (forward 5’-CGGGAAGGGAGAAGACACT-3’ and reverse 5’-GAGTTCCTCACGCCAACG-3’), *Pdcd1l1* (forward 5’-GCTCCAAAGGACTTGTACGTG-3’ and reverse 5’-TGATCTGAAGGGCAGCATTTC-3’), *Arg1* (forward 5’-CTCCAAGCCAAAGTCCTTAGAG-3’ and reverse 5’- AGGAGCTGTCATTAGGGACATC-3’), *Il10* (forward 5’-CTTACTGACTGGCATGAGGATCA-3’ and reverse 5’-GCAGCTCTAGGAGCATGTGG-3’) and *Tgfb1* (forward 5’- ATGTCACGGTTAGGGGCTC-3’ and reverse 5’-GGCTTGCATACTGTGCTGTATAG-3’).

### Public Database Gene Expression Analysis

Normalized gene expression data from the TCGA and project were downloaded from the gene expression profiling interactive analysis (GEPIA).

## Results

### Interleukin-1β Induces pSTAT3 (Y705) in Human Metastatic Melanoma Cells

**Figure 1:**
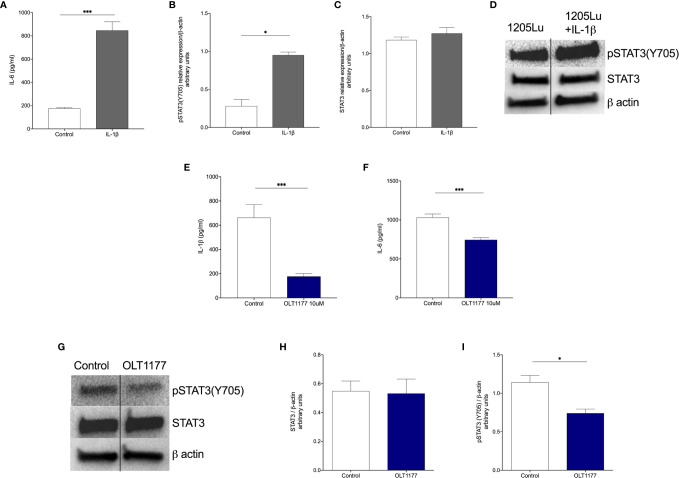
Interkeukin-1β induces pSTAT3(Y705) in human melanoma cells. **(A)** Mean ± SEM of IL-6 production from 1205Lu cells stimulated with IL-1β after 24 hours (N=3). **(B)** Mean ± SEM of STAT3/β-actin ratio for 1205Lu cells shown in **(A)** (N=3). **(C)** Mean ± SEM of pSTAT3(Y705)/β-actin ratio for 1205Lu cells shown in **(A)** (N=3). **(D)** Representative western blot images from **(B, C)**. **(E)** Mean ± SEM of IL-1β production from unstimulated 1205Lu cells treated with OLT1177 after 48 hours (N=3). **(F)** Mean ± SEM of IL-6 production from unstimulated 1205Lu cells treated with OLT1177 after 48 hours (N=3). **(G)** Representative western blot images from **(H, I)**. **(H)** Mean ± SEM of STAT3/β-actin ratio for 1205Lu cells shown in **(G)** (N=3). **(I)** Mean ± SEM of pSTAT3(Y705)/β-actin ratio for 1205Lu cells shown in **(G)** (N=3). *p < 0.05, ***p < 0.001.

We have previously shown that metastatic melanoma cells display constitutively active NLRP3 resulting in spontaneous IL-1β production and release (16). Thus, we sought to assess whether tumor-derived IL-1β induces the IL-6/STAT3 signaling axis in melanoma cells. As shown in **Figure 1A**, we found that stimulation of the human metastatic melanoma 1205Lu cells with recombinant IL-1β increased IL-6 after 24 hours compared to the vehicle-treated control cells (*p*<0.001). Next, we assessed the effect of IL‐1β on STAT3 activation. IL-1β significantly increased pSTAT3(Y705) (**Figures 1B, D**), whereas there was no induction of total STAT3 in the same cells (**Figures 1C, D**). We then determined whether reduction in IL‐1β production *via* NLRP3 inhibition influenced IL‐6 production and STAT3 phosphorylation. For this experiment, we used the synthetic, small molecule OLT1177, a specific NLRP3 inhibitor, which is safe and effective in humans (37, 38). Spontaneous IL‐1β production in 1205Lu cells was reduced 74% with NLRP3 inhibition by OLT1177 at 48 hours, confirming our previously published data (16) (**Figure 1E**, *p*<0.001). The inhibition of NLRP3 activation and the subsequent suppression of IL‐1β production resulted in a 28% reduction in IL‐6 production (**Figure 1F**, *p*<0.001). Analysis of the same cell lysates revealed a significant decrease in Y705 phosphorylation of STAT3 (*p*<0.05) without affecting the total levels of the transcription factor (**Figures 1G–I**). These results were confirmed in another human metastatic melanoma cell line, A357. Consistently, **Supplemental Figures 1A–I** show that IL-1β induction of IL-6/STAT3 axis is indeed consistent amongst different metastatic melanoma cell lines.

These findings reveal that NLRP3‐dependent, tumor-derived IL-1β induces autocrine IL-6 production, which leads to activation of canonical/nuclear functions of STAT3.

### NLRP3 Inhibition Disrupts IL-1β-Induced IL-6/STAT3 Signaling in the Tumor Micro-Environment

**Figure 2:**
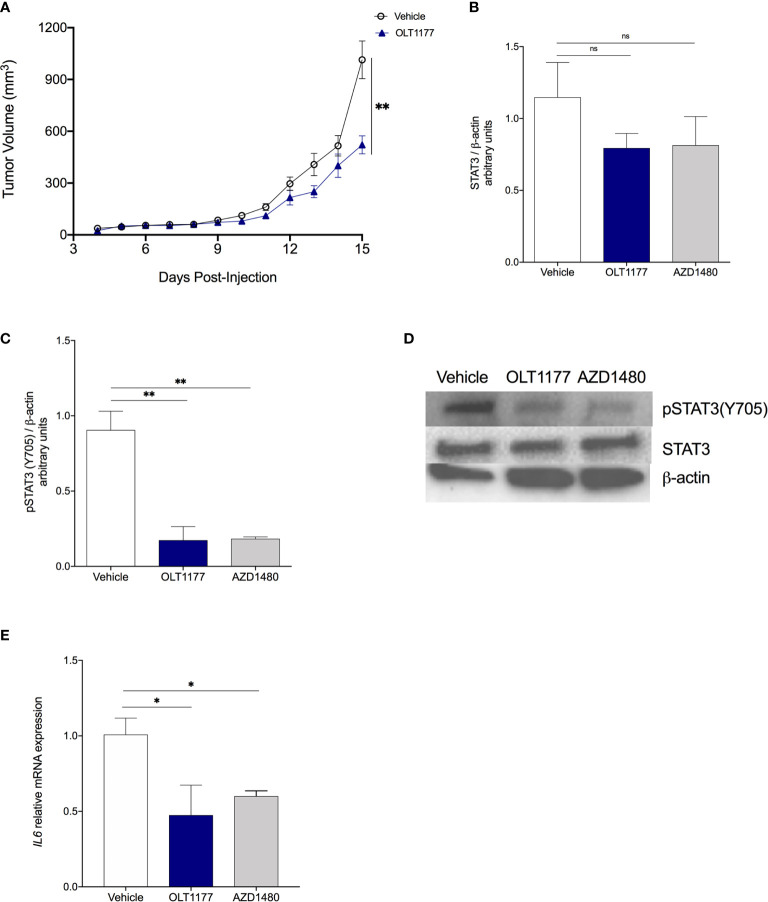
NLRP3 inhibition disrupts IL-1β-induced IL-6/STAT3 signaling in the TME. **(A)** Tumor growth in mice treated with saline control (Vehicle) or OLT1177 (OLT1177) (N=8/group). **(B)** Mean ± SEM of STAT3/β-actin ratio in primary tumors of mice described in **(A)** (N=6). **(C)** Mean ± SEM of pSTAT3(Y705)/β-actin ratio in primary tumors of mice described in **(A)** (N=6). **(D)** Representative western blot images from **(C, D)**. **(E)** Mean ± SEM of relative mRNA expression *Il6* from primary tumors. ns (not significant), *p < 0.05, **p < 0.01.

To confirm our *in vitro* findings *in vivo*, we used the murine melanoma cell line B16F10. Briefly, mice were implanted with B16F10 and on day 5 daily oral treatment of OLT1177 or saline, in the control group, was started. As shown in **Figure 2A**, tumor size was reduced by 49% compared to vehicle treated mice (*p*<0.01). These data are in line with our previous reports, mice treated with OLT1177 revealed a 2-fold reduction in tumor volume compared to vehicle (*p*<0.01) (16). As shown in **Supplement Figure 2A**, daily treatment with the non-specific JAK inhibitor, AZD1480, exhibited a similar reduction in tumor growth when compared to vehicle. Western blot analysis of the primary tumors revealed that treatment with either OLT1177 or AZD1480 reduced constitutive STAT3 levels by 31% and 29%, respectively, but did not reach statistical significance (**Figure 2B**). In contrast, there was a marked and highly significant (*p*<0.01) reduction in Y705 phosphorylated STAT3 by OLT1177 (82%) and AZD1480 (80%) compared to vehicle (**Figures 2C, D**). No changes in Y705 phosphorylation of STAT3 were observed between OLT1177 or AZD1480 treatments (**Figures 2C, D**).

We next examined the JAK-dependent, IL‐6/STAT3 axis controlled by NLRP3. Genetic analysis of tumors from mice treated with OLT1177 or AZD1480 showed reduced gene expression of *Il6* in comparison to the control group (both *p*<0.05) (**Figure 2E**). In humans, analyses of the TCGA dataset revealed positive mRNA correlations from cutaneous melanoma biopsies for expression of NLRP3 and IL‐6 (p=4.86e^-20^), IL‐6 and the STAT3 target gene Socs3 (p=1.82e^-62^), IL-1β and Socs3 (p=1.71e^-31^) (**Supplemental Figures 2B–D**). Overall, these data confirm the *in vitro* observation and suggest the induction of IL‐6/STAT3 following NLRP3 inflammasome activation as an integral driver of melanoma progression.

### Tumor-NLRP3 Drives IL-6/STAT3 Signaling in the Bone Marrow

**Figure 3:**
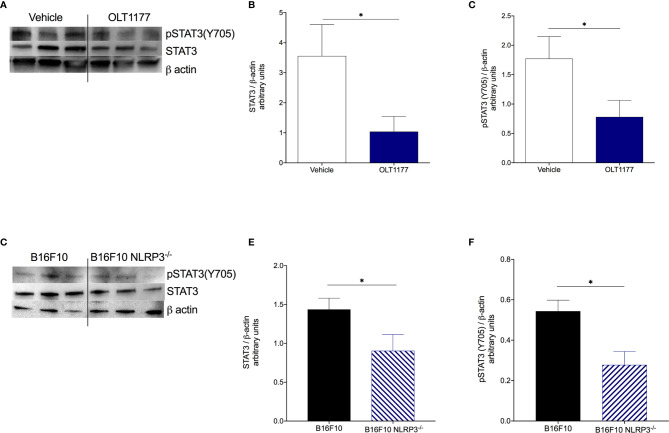
Tumor-NLRP3 drives IL-6/STAT3 signaling in the bone marrow. **(A–C)** Mice were implanted with B16F10 and treated with saline control (Vehicle) or OLT1177 (OLT1177), on day 15 bone marrow was assessed *via* western blot. **(A)** Representative western blot images from **(B, C)**. **(B)** Mean ± SEM of STAT3/β-actin ratio in bone marrow of mice described in **(A–C)** (N=6). **(C)** Mean ± SEM of pSTAT3(Y705)/β-actin ratio in bone marrow of mice described in **(A–C)** (N=6). **(D–F)** Mice were implanted with B16F10 or B16F10 NLRP3^-/-^ cells, on day 15 bone marrow was assessed *via* western blot. **(D)** Representative western blot images from **(E, F)**. **(E)** Mean ± SEM of STAT3/β-actin ratio in bone marrow of mice described in **(D–F)** (N=6). **(F)** Mean ± SEM of pSTAT3(Y705)/β-actin ratio in bone marrow of mice described in **(D–F)** (N=6). *p < 0.05.

We previously observed that tumor NLRP3-dependent IL-1β production increases bone marrow production of both IL-1β and IL-6 (16). Therefore, we sought to assess whether tumor-associated NLRP3 activity also affects the IL-1β/IL-6/STAT3 axis in host myeloid cells. To investigate this, mice were implanted with B16F10 and treated as described in **Figure 2A**. On day 15 when tumors are in exponential growth, bone marrow cells were analyzed by Western blot. As shown in **Figures 3A–C**, tumor-bearing mice receiving OLT1177 exhibited significantly less constitutive STAT3 (*p*<0.05) and pSTAT3(Y705) (*p*<0.05) in the bone marrow compared to vehicle-treated mice. Next, we confirmed the role of tumor-derived NLRP3 by implanting wild-type B16F10 or B16F10 deficient of NLRP3 (B16F10 *nlrp3^-/-^*). After 15 days, we analyzed bone marrow-derived cells as described above. As shown in **Figures 3D–F**, bone marrow-derived cells from mice implanted with B16F10 *nlrp3^-/-^* cells revealed significantly less total STAT3 (*p*<0.05) and pSTAT3(Y705) (*p*<0.05) compared to the cells isolated from mice implanted with wild-type B16F10. In addition to our published data (16), these results posit NLRP3 as a key driver of STAT3 in melanoma-associated inflammation.

### pSTAT3(Y705) Regulates PMN-MDSCs Immunosuppressive Gene Expression

**Figure 4:**
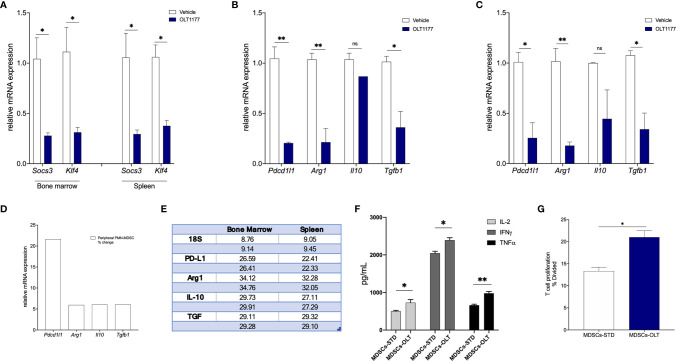
pSTAT3(Y705) regulates PMN-MDSCs immunosuppressive gene expression. Mice were implanted with B16F10 and treated with saline control (Vehicle) or OLT1177 (OLT1177), on day 15 PMN-MDSCs were isolated from bone marrow and spleen. **(A)** Mean ± SEM of relative mRNA expression of *Socs3* and *Klf4* from PMN-MDSCs isolated from bone marrow or spleen of tumor-bearing mice described above (N=2, pooled from 4 mice/group. 2 independent experiments). **(B)** Mean ± SEM of relative mRNA expression of *Pdcd1l1*, *Arg1, Il10* and *Tgfb1* from PMN-MDSCs isolated from bone marrow of mice described above (N=2, pooled from 4 mice/group. 2 independent experiments). **(C)** Mean ± SEM of relative mRNA expression of *Pdcd1l1*, *Arg1, Il10* and *Tgfb1* from PMN-MDSCs isolated from spleen of mice described above (N=2, pooled from 4 mice/group. 2 independent experiments). **(D)** Fold change of relative mRNA expression of *Pdcd1l1*, *Arg1, Il10* and *Tgfb1* from PMN-MDSCs isolated from spleen compared to PMN-MDSCs isolated from bone marrow. **(E)** Cq values (in duplicate) depicting raw rtPCR data described in **(D)**. **(E)** Co-culture of naïve CD8+ T cells with MDSCs isolated from tumor-bearing mice treated with saline control (Vehicle) or OLT1177 (OLT1177). **(F)** Mean ± SEM for IL-2, IFNγ and TNFα production from co-culture described in **(E)** (N=3). **(G)** T cell proliferation from co-culture described in **(F)** (N=3). ns (not significant), *p < 0.05, **p < 0.01.

Inhibition of tumor-derived NLRP3 results in reduced PMN-MDSCs expansion from the bone marrow and less infiltrating MDSCs in the TME (16). Thus, we sought to determine if the reduction in IL-6/STAT3 activation observed following NLRP3 inhibition (**Figure 3**) affects expression of genes associated with the immunosuppressive activity of PMN-MDSCs. Mice were implanted with B16F10 and on day 5 treated with daily oral OLT1177 or saline. PMN-MDSCs from tumor-bearing mice treated with vehicle or OLT1177 were isolated from the bone marrow and spleen using flow cytometry and assessed for gene expression (**Supplemental Figure 3**). As shown in **Figure 4A**, bone marrow and spleen-derived PMN-MDSCs from mice treated with OLT1177 revealed a substantial reduction in the expression of genes associated with nuclear STAT3 activity, namely, *Socs3* and *Klf4*, confirming reduced STAT3 activity. These same cells were also assessed for the expression of immunosuppressive genes, such as *pdcd1l1* (PD-L1), *Arg1* (Arginase 1), *Il10* (IL-10) and *Tgfb1* (TGF-β1). With the exception of *Il10*, these genes were significantly reduced in the bone marrow of mice treated with OLT1177, for example, *Pdcd1l1* (86%, *p*<0.01), *Arg1* (80%, *p*<0.01) and *Tgfb1* (65*%, p<0.05*) (**Figure 4B**). In the spleen (**Figure 4C**), we also observed significant decreased levels of *Pdcd1l1* (74%, *p*<0.01), *Arg1* (83%, *p*<0.01) and *Tgfb1* (69%*, p<0.05*) as well as a non-significant decrease in, *Il10*. To confirm the role of STAT3 in these NLRP3-mediated changes of PMN-MDSCs, tumor-bearing mice were treated with AZD1480 and PMN-MDSCs were assessed as described above. As shown in **Supplemental Figures 4A, B**, PMN-MDSCs obtained from AZD1480-treated mice exhibited decreased immunosuppressive gene expression compared to vehicle, similar to that observed in OLT1177-treated mice. We next compared relative gene expression from bone marrow-derived PMN-MDSCs to spleen-derived PMN-MDSCs. **Figures 4D, E** depicts fold-change increases in immunosuppressive gene expression upon migration in the peripherical tissues as well as relative Cq values. Overall, these data show that inhibiting tumor-NLRP3 alone is sufficient to reduce IL-6/STAT3 activation, resulting in the reduction of immunosuppressive gene expression in PMN-MDSCs.

We next examined the effect of NLRP3 inhibition on MDSCs function in *ex vivo* conditions. Briefly, naïve CD8+ T cells were isolated from non-tumor-bearing mice and co-cultured with spleen MDSCs isolated from tumor-bearing mice fed standard or OLT1177 diet. On day 3, T cell proliferation was determined using flow cytometry and supernatants were assessed for T cell associated cytokines. **Figure 4F** depicts the levels of IL-2, IFNγ and TNFα secretion from the co-cultures described above. Supernatants from co-cultures containing MDSCs isolated from mice treated with OLT1177 revealed significantly higher levels of IL-2 (*p*<0.05), IFNγ (*p*<0.05) and TNFα (*p*<0.01) compared to co-cultures containing MDSCs from mice fed the standard diet (**Figure 4F**). Consistently, T cell proliferation was significantly higher in co-cultures containing MDSCs isolated from mice treated with OLT1177 (**Figure 4G**). Taken together, these data suggest tumor-NLRP3 as a potential target to suppress MDSCs, which ultimately dampen cytotoxic T cell functions.

## Discussion

**Figure 5:**
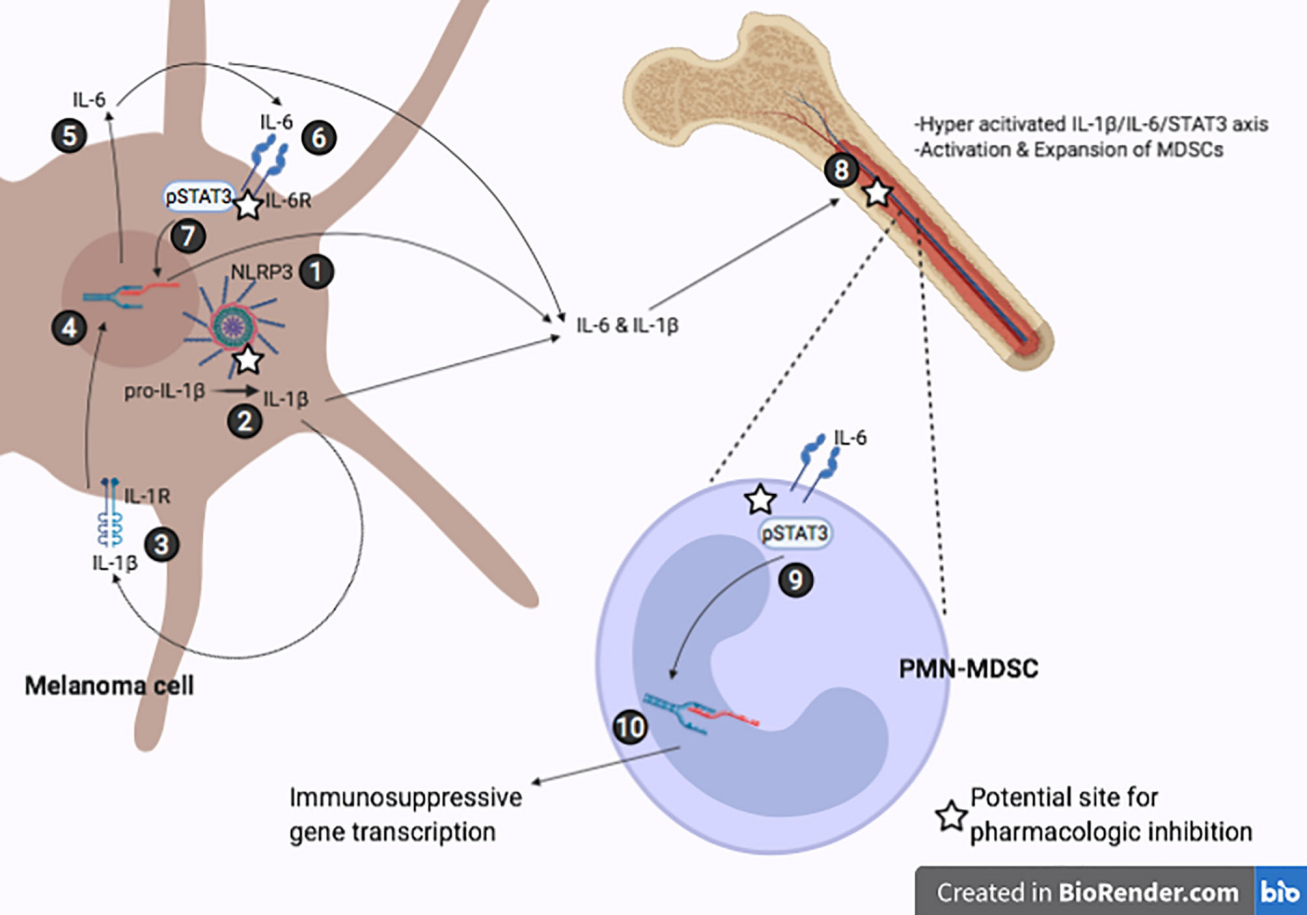
Representative IL-1β/IL-6/STAT3 axis in melanoma induced immunosuppression. (1) Constitutively active NLRP3 inflammasome. (2) NLRP3-mediated cleavage of pro-IL-1β to mature IL-1β. (3-4) Secreted IL-1β binds the IL-1R receptor leading to increased IL-6 transcription. (5-6) IL-6 secreted by melanoma cell binds IL-6R. (7) JAK/STAT3 signaling cascade leads to phosphorylation of STAT3 and induces transcriptional activity leading to more IL-6 production. (8) Tumor-derived IL-1β and IL-6 travel to bone marrow and promote hyperactivation of IL-1β/IL-6/STAT3, further inducing melanoma-associated inflammation and activation of MDSCs. (9-10) IL-6 signaling in PMN-MDSCs results in nuclear localization of STAT3 and upregulation of immunosuppressive genes.

Although it is well established that tumor-induced inflammation drives immunosuppression (4, 8), specific molecular mechanism(s) that account for the inflammation are not fully understood. In this study, we elucidated a novel mechanism that demonstrates how NLRP3 activity in melanoma drives inflammation resulting in increased expression of immunosuppressive genes in MDSCs. Using a mouse model of melanoma, we report here that NLRP3 activation induces IL‐1β-mediated IL‐6 production resulting in STAT3 activation. We also observed that with specific inhibition of NLRP3, both tumor volume and STAT3 signaling in the TME are significantly reduced. These observations are consistent with the concept of tumor-derived inflammation promoting tumor progression and that IL-1β induces IL-6/STAT3 activation in melanoma. As depicted in **Figure 5** and in line with our previous findings, tumor-derived constitutive NLRP3 activity induces IL-1β and IL-6 in bone marrow and spleen cells (16), we show that tumor-bearing mice treated with OLT1177 reveal reductions in both total STAT3 as well as pSTAT3 (Y705) in bone marrow cells. We conclude an NLRP3-mediated autoinflammatory loop drives IL-6/STAT3 regulated immunosuppressive gene expression. Thus, NLRP3 is a therapeutic target to reverse canonical STAT3 mediated cancer promotion.

The implication of tumor-derived NLRP3 driving IL‐1β-IL‐6-STAT3 signaling was confirmed in mice implanted with NLRP3 deficient B16F10 cells (B16F10 *nlrp3^-/-^*). Using NLRP3 deficient B16F10 cells, we observed similar reductions of STAT3 and pSTAT3 (Y705) comparable to those of OLT1177 treatment. Upon knock-down of NLRP3 in the tumor, IL-1β processing is arrested and, therefore mature IL-1β-induced IL-6/STAT3 axis is no longer being amplified. With reduced IL‐1β activity, there is less tumor-derived IL-1β and IL-6 activity on bone marrow cells and ultimately decreased STAT3 activity in this cell population (**Figure 5**), supporting our previous findings that tumor-NLRP3 activity induces IL-1β and IL-6 in the bone marrow (16).

Linking tumor-NLRP3 induction of IL-6/STAT3 to immunosuppression, we assessed PMN-MDSCs in bone marrow and spleen from tumor-bearing mice treated with OLT1177. Disruption of STAT3 transcriptional activity was associated with the reduction of STAT3 target genes *Socs3* and *Klf4*. Mice treated with OLT1177 revealed the IL‐1β-dependent role of STAT3 in promoting the levels of immunosuppressive genes. We show marked reductions in *Pdcd1l1*, *Arg1*, *Il10* and *Tfgb1* in PMN-MDSCs from bone marrow and spleen of tumor-bearing mice. In order to show the role of JAK-mediated STAT3 phosphorylation in PMN-MDSCs, we treated mice with AZD1480 and observed similar reductions in gene expression as we observed with OLT1177 treatment. These findings implicate IL-1β-dependent IL-6/STAT3 signaling as an integral driver of tumor-induced inflammation that promotes immunosuppressive gene expression of PMN-MDSCs. These data also provide new insights into therapeutic approaches to disrupt tumor immune evasion offered by NLRP3 inhibitors in melanoma (**Figure 5**).

Cytokines produced by tumors change not only immune cell function in the TME, but also the expansion and activation of myeloid cells in the bone marrow (19, 29, 39). We recently reported that constitutively active NLRP3 in melanoma cells induces IL-1β and IL-6 in the host, resulting in the expansion of MDSCs (16). In line with those findings, in the present study we demonstrate that elevated IL-6 induces STAT3 activation, which is an established pro-tumor mechanism present in many cancers for MDSC activation as well as gene expression (32, 36). Of note, other STATs have been implicated in melanoma progression, such as STAT5 (40). This prior study showed activity of STAT5 was mediated in park by JAK1, of which IL-6 is a known activator, therefore we cannot rule out other STAT activity. Notably, activation of STAT3 in cancer has mostly been attributed to loss of function of STAT3 regulatory genes, but not by increased activity of upstream inducers of IL-6 such as NLRP3 (36, 41, 42). Here, we link NLRP3 activity in melanoma cells to the IL-1β induced-IL‐6/STAT3 axis, thus providing an additional mechanism to target STAT3 signaling in melanoma. Our study also expands on this pathway in that NLRP3 inhibition reduces transcriptional activity of STAT3; thus we observed markedly lower gene expression in PMN-MDSCs, namely: *Pdcd1l1* (PD-L1), *Arg1*, *Il10* and *Tgfb1*. Moreover, NLRP3 inhibition reduced the immunosuppressive potential as shown by increased T cell proliferation and production IL-2, IFNγ and TNFα in co-culture. These findings suggest PMN-MDSCs are reliant on tumor inflammatory signals for upregulation of immunosuppressive genes, which in turn, dictate function.

This concept is of particular importance for immunotherapy. Levels of MDSCs have been proposed as a predictive marker for response to immunotherapy (43, 44). Specifically, in melanoma *Pdcd1l1*, the gene encoding Programed Death-Ligand1 (PD-L1), is highly expressed in MDSCs and elevated levels of circulating MDSCs may serve as an indicator of checkpoint inhibition and reduced tumor growth (44–47). However, a recent study reported that anti-PD-1 therapy increased melanoma-NLRP3 activity, resulting in increased PD-L1 expression in the tumor (48). Nevertheless, upon knockdown of tumor-derived NLRP3, tumor progression markedly slowed with anti-PD-1, resulting in decreased PMN-MDSC infiltration into the TME (47). Alternatively, we observed NLRP3 activation in B16F10 metastatic melanoma independent of anti-PD-1 therapy. Importantly, a consequence of NLRP3 activation in the tumor are alterations in the systemic host immune response, a concept that is highly relevant in immunotherapeutic approaches targeting inflammatory tumors. Here, we report how tumor-NLRP3 activity promotes IL-6/STAT3 signaling in the bone marrow, which then regulates immunosuppressive gene expression in PMN-MDSCs resulting in a defunct cell population.

As STAT3 phosphorylation by the JAK1/2 is well known in cancer, we used AZD1480, which suppress JAK1/2 (49, 50), to demonstrate that phosphorylation of STAT3 at Y705 is dependent of JAK1/2. We show the reduction in pSTAT3 (Y705) in **Figure 2** and observed a significant reduction in B16F10 tumor growth in mice treated with AZD1480 (**Supplemental Figure 1A**). Several JAK1/2 inhibitors are used to treat autoimmune diseases such as rheumatoid arthritis, psoriasis, psoriatic arthritis and ulcerative colitis. Tofacitinib, ruxolitinib, baricitinib and upadacitinib are JAK1/2 inhibitors also used in alopecia areata (51, 52). JAK1/2 inhibitors reduce several cytokines; among these are IL-2, IL-15, IL‐12/23 IL‐6, IFNγ, IFNα, IL‐10 and G‐CSF. Upon binding to their respective type 1 and type 2 receptors, JAKs are recruited to the cytosolic domains resulting in the phosphorylation of STATs, initiation of transcription and subsequent biological activities of the respective cytokines. In human peripheral blood mononuclear cells from healthy donors, JAK inhibitors added *in vitro* reduced STAT3 (Y705) phosphorylation (53). Although the benefit of reducing STAT3 phosphorylation at Y705 results in suppression of transcriptional activation of several cytokines in autoimmune diseases, this mechanism is also valid in treatment of cancer. For example, myeloproliferative neoplasms such as chronic lymphocytic leukemia (54) and solid tumors such as colorectal cancer (55) are treated with JAK inhibitors. In patients with autoimmune diseases or cancer, JAK inhibitors are associated with serious side effects such as anemia, nausea, vomiting, weight loss, myalgia, herpes zoster, neutropenia and low levels of circulating lymphocytes. JAK inhibitors are also a risk for life-threatening thromboembolic events such as deep venous thrombosis and pulmonary embolism (56). Upadacitinib treatment in rheumatoid arthritis resulted in hepatic dysfunction in 7.6% of the patients (57). By comparison, patients treated with oral OLT1177 for gout flares (38) or heart failure (58) report no side effects but rather an increase in well-being.

Altogether, these findings reveal how metastatic melanoma cells amplify IL-6 signaling through an NLRP3-mediated autoinflammatory loop, which in turn, drives IL-6/STAT3 regulated immunosuppressive gene expression in PMN-MDSCs. The proposed pathway elicits NLRP3 as a therapeutic target to reverse canonical STAT3 mediated mechanisms of cancer promotion.

## Data Availability Statement

The raw data supporting the conclusions of this article will be made available by the authors, without undue reservation.

## Ethics Statement

The animal study was reviewed and approved by University of Colorado Animal Care and Use Committee.

## Author Contributions

IT designed the research. IT, AD, MB and NP carried out the research. IT, AD, CM, and CD, analyzed the data. LJ critically read the paper. IT, CM, and CD wrote the paper. All authors contributed to the article and approved the submitted version.

## Funding

These studies are supported by NIH Grant AI-15614 (to CD), the Interleukin Foundation, and Olatec Industries. The funder was not involved in the study design, collection, analysis, interpretation of data, the writing of this article or the decision to submit it for publication.

## Conflict of Interest

LJ serves on Olatec’s Scientific Advisory Board and receives compensation. CD serves as Chairman of Olatec’s Scientific Advisory Board, is co-Chief Scientific Officer, receives compensation and has equity in Olatec. CM serves as Director for Olatec’s Innovative Science Program and has equity in Olatec.

The remaining authors declare that the research was conducted in the absence of any commercial or financial relationships that could be construed as a potential conflict of interest.

## Publisher’s Note

All claims expressed in this article are solely those of the authors and do not necessarily represent those of their affiliated organizations, or those of the publisher, the editors and the reviewers. Any product that may be evaluated in this article, or claim that may be made by its manufacturer, is not guaranteed or endorsed by the publisher.

## Supplementary Material

Supplementary Figure 1 **(A)** Mean ± SEM of IL-6 production from A357 cells stimulated with IL-1β after 24 hours (N=3). **(B)** Mean ± SEM of STAT3/β-actin ratio for 1205Lu cells shown in **(A)** (N=3). **(C)** Mean ± SEM of pSTAT3(Y705)/β-actin ratio for A357 cells shown in (a) (N=3). **(D)** Representative western blot images from **(B, C)**. **(E)** Mean ± SEM of IL-1β production from unstimulated A357 cells treated with OLT1177 after 48 hours (N=3). **(F)** Mean ± SEM of IL-6 production from unstimulated A357 cells treated with OLT1177 after 48 hours (N=3). **(G)** Representative western blot images from **(H)** and **(I)**. **(H)** Mean ± SEM of STAT3/β-actin ratio for A357 cells shown in **(G)** (N=3). **(I)** Mean ± SEM of pSTAT3(Y705)/β-actin ratio for A357 cells shown in **(G)** (N=3). **p* < 0.05, ****p* < 0.001.

Supplementary Figure 2 **(A)**Tumor growth in mice treated with saline control (Vehicle) or AZD1480 (AZD1480) (N=8/group). **(B)** Correlation between NLRP3 and IL‐6 expression in human cutaneous melanoma (SKCM) TCGA datasets (N=363). **(C)** Correlation between of IL-6 and SOCS3 expression in human cutaneous melanoma (SKCM) TCGA datasets (N=363). **(D)** Correlation between of SOCS3 and IL-1β expression in human cutaneous melanoma (SKCM) TCGA datasets (N=363).

Supplementary Figure 3 FACs gating strategy depicting PMN-MDSCs populations that were isolated bone marrow of tumor-bearing mice.

Supplementary Figure 4 Mice were implanted with B16F10 and treated with saline control (Vehicle) or AZD1480 (AZD1480), on day 15 PMN-MDSCs were isolated from bone marrow and spleen. **(A)** Mean ± SEM of relative mRNA expression of *Pdcd1l1*, *Arg1, Il10* and *Tgfb1* from PMN-MDSCs isolated from bone marrow of mice described above (N=2, pooled from 4 mice/group. 2 independent experiments). **(B)** Mean ± SEM of relative mRNA expression of *Pdcd1l1*, *Arg1, Il10* and *Tgfb1* from PMN-MDSCs isolated from bone marrow of mice described above (N=2, pooled from 4 mice/group. 2 independent experiments). **p* < 0.05, ***p* < 0.001, ****p* < 0.001.

## References

[1] Bi J Tian Z. NK Cell Exhaustion. Front Immunol (2017) 8:760. 10.3389/fimmu.2017.00760 28702032 PMC5487399

[2] McLane LM Abdel-Hakeem MS Wherry EJ. CD8 T Cell Exhaustion During Chronic Viral Infection and Cancer. Annu Rev Immunol (2019) 37:457–95. 10.1146/annurev-immunol-041015-055318 30676822

[3] Di Gennaro P Gerlini G Caporale R Sestini S Brandani P Urso C. T Regulatory Cells Mediate Immunosuppresion by Adenosine in Peripheral Blood, Sentinel Lymph Node and TILs From Melanoma Patients. Cancer Lett (2018) 417:124–30. 10.1016/j.canlet.2017.12.032 29306022

[4] Osborne DG Domenico J Luo Y Reid AL Amato C Zhai Z. Interleukin-37 Is Highly Expressed in Regulatory T Cells of Melanoma Patients and Enhanced by Melanoma Cell Secretome. Mol Carcinog (2019) 58:1670–9. 10.1002/mc.23044 PMC6692223 31099111

[5] Wei SC Duffy CR Allison JP. Fundamental Mechanisms of Immune Checkpoint Blockade Therapy. Cancer Discovery (2018) 8:1069–86. 10.1158/2159-8290.CD-18-0367 30115704

[6] Solinas G Germano G Mantovani A Allavena P. Tumor-Associated Macrophages (TAM) as Major Players of the Cancer-Related Inflammation. J Leukoc Biol (2009) 86:1065–73. 10.1189/jlb.0609385 19741157

[7] Veglia F Perego M Gabrilovich D. Myeloid-Derived Suppressor Cells Coming of Age. Nat Immunol (2018) 19:108–19. 10.1038/s41590-017-0022-x PMC5854158 29348500

[8] Chen J Ye Y Liu P Yu W Wei F Li H. Suppression of T Cells by Myeloid-Derived Suppressor Cells in Cancer. Hum Immunol (2017) 78:113–9. 10.1016/j.humimm.2016.12.001 27939507

[9] Elkabets M Ribeiro VSG Dinarello CA Ostrand-Rosenberg S Di Santo JS Apte RN. IL-1beta Regulates a Novel Myeloid-Derived Suppressor Cell Subset That Impairs NK Cell Development and Function. Eur J Immunol (2010) 40:3347–57. 10.1002/eji.201041037 PMC3373225 21110318

[10] Fujimura T Kambayashi Y Aiba S. Crosstalk Between Regulatory T Cells (Tregs) and Myeloid Derived Suppressor Cells (MDSCs) During Melanoma Growth. Oncoimmunology (2012) 1:1433–4. 10.4161/onci.21176 PMC3518528 23243619

[11] Ballbach M Dannert A Singh A Siegmund DM Handgretinger R Piali L. Expression of Checkpoint Molecules on Myeloid-Derived Suppressor Cells. Immunol Lett (2017) 192:1–6. 10.1016/j.imlet.2017.10.001 28987474

[12] Umansky V Blattner C Gebhardt C Utikal J. The Role of Myeloid-Derived Suppressor Cells (MDSC) in Cancer Progression. Vaccines (Basel) (2016) 4(4):36. 10.3390/vaccines4040036 PMC5192356 27827871

[13] Hoejberg L Bastholt L Johansen JS Fode K Schmidt H. Serum Interleukin-6 as a Prognostic Biomarker in Patients With Metastatic Melanoma. Melanoma Res (2012) 22:287–93. 10.1097/CMR.0b013e3283550aa5 22617301

[14] Jiang H Gebhardt C Umansky L Bechkove P Schulze TJ Utikal J. Elevated Chronic Inflammatory Factors and Myeloid-Derived Suppressor Cells Indicate Poor Prognosis in Advanced Melanoma Patients. Int J Cancer (2015) 136:2352–60. 10.1002/ijc.29297 25353097

[15] Tobin RP Jordan KR Kapoor P Spongberg E Davis D Vorwald VM. IL-6 and IL-8 Are Linked With Myeloid-Derived Suppressor Cell Accumulation and Correlate With Poor Clinical Outcomes in Melanoma Patients. Front Oncol (2019) 9:1223. 10.3389/fonc.2019.01223 31781510 PMC6857649

[16] Tengesdal I Menon DR Osborne DG Neff CP Powers NE Gamboni F. Targeting Tumor-Derived NLRP3 Reduces Melanoma Progression by Limiting MDSCs Expansion. Proc Natl Acad Sci USA (2021). In press, Targeting tumor-derived NLRP3 reduces melanoma progression by limiting MDSCs expansion. Proc Natl Acad Sci USA (2021). 10.1073/pnas.2000915118 PMC7958415 33649199

[17] Lippitz BE Harris RA. Cytokine Patterns in Cancer Patients: A Review of the Correlation Between Interleukin 6 and Prognosis. Oncoimmunology (2016) 5:e1093722. 10.1080/2162402X.2015.1093722 27467926 PMC4910721

[18] Liu H Ren G Wang T Chen Y Gong C Bai Y. Aberrantly Expressed Fra-1 by IL-6/STAT3 Transactivation Promotes Colorectal Cancer Aggressiveness Through Epithelial-Mesenchymal Transition. Carcinogenesis (2015) 36:459–68. 10.1093/carcin/bgv017 PMC4392608 25750173

[19] Weber R Riester Z Hüser L Sticht C Siebenmorgen A Groth C. IL-6 Regulates CCR5 Expression and Immunosuppressive Capacity of MDSC in Murine Melanoma. J Immunother Cancer (2020) 8. 10.1136/jitc-2020-000949 PMC7422659 32788238

[20] Wu ZS Cheng XW Wang XN Song NJ. Prognostic Significance of Phosphorylated Signal Transducer and Activator of Transcription 3 and Suppressor of Cytokine Signaling 3 Expression in Human Cutaneous Melanoma. Melanoma Res (2011) 21:483–90. 10.1097/CMR.0b013e32834acc37 21876460

[21] Kusaba T Nakayama T Yamazumi K Yakata Y Yoshizaki A Inoue K. Activation of STAT3 Is a Marker of Poor Prognosis in Human Colorectal Cancer. Oncol Rep (2006) 15:1445–51. 10.3892/or.15.6.1445 16685378

[22] Chen Y Wang J Wang X Li H Lv Q Zhu J. STAT3, a Poor Survival Predicator, Is Associated With Lymph Node Metastasis From Breast Cancer. J Breast Cancer (2013) 16:40–9. 10.4048/jbc.2013.16.1.40 PMC3625768 23593080

[23] Huynh J Chand A Gough D Ernst M. Therapeutically Exploiting STAT3 Activity in Cancer - Using Tissue Repair as a Road Map. Nat Rev Cancer (2019) 19:82–96. 10.1038/s41568-018-0090-8 30578415

[24] Langlais D Couture C Balsalobre A Drouin J. The Stat3/GR Interaction Code: Predictive Value of Direct/Indirect DNA Recruitment for Transcription Outcome. Mol Cell (2012) 47:38–49. 10.1016/j.molcel.2012.04.021 22633955

[25] Kortylewski M Kujawski M Wang T Wei S Zhang S Pilon-Thomas S. Inhibiting Stat3 Signaling in the Hematopoietic System Elicits Multicomponent Antitumor Immunity. Nat Med (2005) 11:1314–21. 10.1038/nm1325 16288283

[26] Kortylewski M Yu H. Role of Stat3 in Suppressing Anti-Tumor Immunity. Curr Opin Immunol (2008) 20:228–33. 10.1016/j.coi.2008.03.010 PMC2488961 18479894

[27] Yu H Pardoll D Jove R. STATs in Cancer Inflammation and Immunity: A Leading Role for STAT3. Nat Rev Cancer (2009) 9:798–809. 10.1038/nrc2734 19851315 PMC4856025

[28] Lin Y Yang X Liu W Li B Yin W Shi Y. Chemerin has a Protective Role in Hepatocellular Carcinoma by Inhibiting the Expression of IL-6 and GM-CSF and MDSC Accumulation. Oncogene (2017) 36:3599–608. 10.1038/onc.2016.516 28166197

[29] Hart KM Byrne KT Molloy MJ Usherwood EM Berwin B. IL-10 Immunomodulation of Myeloid Cells Regulates a Murine Model of Ovarian Cancer. Front Immunol (2011) 2:29. 10.3389/fimmu.2011.00029 22566819 PMC3342001

[30] Chan LC Li CW Xia W Hsu JM Lee HH Cha JH. IL-6/JAK1 Pathway Drives PD-L1 Y112 Phosphorylation to Promote Cancer Immune Evasion. J Clin Invest (2019) 129:3324–38. 10.1172/JCI126022 PMC6668668 31305264

[31] Narita Y Kitamura H Wakita D Sumida K Msauko K Terada S. The Key Role of IL-6-Arginase Cascade for Inducing Dendritic Cell-Dependent CD4(+) T Cell Dysfunction in Tumor-Bearing Mice. J Immunol (2013) 190:812–20. 10.4049/jimmunol.1103797 23248265

[32] Vasquez-Dunddel D Pan F Zeng Q Gorbounov M Albesiano E Fu J. STAT3 Regulates Arginase-I in Myeloid-Derived Suppressor Cells From Cancer Patients. J Clin Invest (2013) 123:1580–9. 10.1172/JCI60083 PMC3613901 23454751

[33] Latz E Xiao TS Stutz A. Activation and Regulation of the Inflammasomes. Nat Rev Immunol (2013) 13:397–411. 10.1038/nri3452 23702978 PMC3807999

[34] Tosato G Jones KD. Interleukin-1 Induces Interleukin-6 Production in Peripheral Blood Monocytes. Blood (1990) 75:1305–10. 10.1182/blood.V75.6.1305.1305 2310829

[35] Broudy VC Kaushansky K Harlan JM Adamson JW. Interleukin 1 Stimulates Human Endothelial Cells to Produce Granulocyte-Macrophage Colony-Stimulating Factor and Granulocyte Colony-Stimulating Factor. J Immunol (1987) 139:464–8. 3298430

[36] Jiang M Chen J Zhang W Zhang R Ye Y Liu P. Interleukin-6 Trans-Signaling Pathway Promotes Immunosuppressive Myeloid-Derived Suppressor Cells *via* Suppression of Suppressor of Cytokine Signaling 3 in Breast Cancer. Front Immunol (2017) 8:1840. 10.3389/fimmu.2017.01840 29326716 PMC5736866

[37] Marchetti C Swartzwelter B Gamboni F Neff CP Richter K Azam T. OLT1177, A Beta-Sulfonyl Nitrile Compound, Safe in Humans, Inhibits the NLRP3 Inflammasome and Reverses the Metabolic Cost of Inflammation. Proc Natl Acad Sci USA (2018) 115:E1530–9. 10.1073/pnas.1716095115 PMC5816172 29378952

[38] Viola Klück M Jansen T Janssen M Comarniceanu A Efdé M Tengesdal IW. Dapansutrile, an Oral Selective NLRP3 Inflammasome Inhibitor, for Treatment of Gout Flares: An Open-Label, Dose-Adaptive, Proof-of-Concept, Phase 2a Trial. Lancet Rheumatol (2020). 10.1016/S2665-9913(20)30065-5 PMC7523621 33005902

[39] Eriksson E Milenova I Wenthe J Moreno R Alemany R Loskog A. IL-6 Signaling Blockade During CD40-Mediated Immune Activation Favors Antitumor Factors by Reducing TGF-Beta, Collagen Type I, and PD-L1/PD-1. J Immunol (2019) 202:787–98. 10.4049/jimmunol.1800717 30617223

[40] Mirmohammadsadegh A Hassan M Bardenheuer W Marini A Gustrau A Nambiar S. STAT5 Phosphorylation in Malignant Melanoma Is Important for Survival and is Mediated Through SRC and JAK1 Kinases. J Invest Dermatol (2006) 126:2272–80. 10.1038/sj.jid.5700385 16741510

[41] Jiang M Zhang WW Liu P Yu W Liu T Yu J. Dysregulation of SOCS-Mediated Negative Feedback of Cytokine Signaling in Carcinogenesis and Its Significance in Cancer Treatment. Front Immunol (2017) 8:70. 10.3389/fimmu.2017.00070 28228755 PMC5296614

[42] Peyser ND Du Y Li H Lui V Xiao X Chan TA. Loss-Of-Function PTPRD Mutations Lead to Increased STAT3 Activation and Sensitivity to STAT3 Inhibition in Head and Neck Cancer. PloS One (2015) 10:e0135750. 10.1371/journal.pone.0135750 26267899 PMC4534317

[43] Martens A Wistuba-Hamprecht K Foppen MG Yuan J Postow MA Wong P. Baseline Peripheral Blood Biomarkers Associated With Clinical Outcome of Advanced Melanoma Patients Treated With Ipilimumab. Clin Cancer Res (2016) 22:2908–18. 10.1158/1078-0432.CCR-15-2412 PMC5770142 26787752

[44] Weide B Martens A Zelba H Stutz C Derhovanessian E Di Giacoma AM. Myeloid-Derived Suppressor Cells Predict Survival of Patients With Advanced Melanoma: Comparison With Regulatory T Cells and NY-ESO-1- or Melan-A-Specific T Cells. Clin Cancer Res (2014) 20:1601–9. 10.1158/1078-0432.CCR-13-2508 24323899

[45] Noman MZ Desantis G Janji B Hasmim M Karray S Dessen P. PD-L1 Is a Novel Direct Target of HIF-1alpha, and Its Blockade Under Hypoxia Enhanced MDSC-Mediated T Cell Activation. J Exp Med (2014) 211:781–90. 10.1084/jem.20131916 PMC4010891 24778419

[46] Meyer C Cagnon L Costa-Nunes CM Baumgaertner P Montandon N Leyvraz L. Frequencies of Circulating MDSC Correlate With Clinical Outcome of Melanoma Patients Treated With Ipilimumab. Cancer Immunol Immunother (2014) 63:247–57. 10.1007/s00262-013-1508-5 PMC11029062 24357148

[47] Hou A Hou K Huang Q Lei Y Chen W. Targeting Myeloid-Derived Suppressor Cell, a Promising Strategy to Overcome Resistance to Immune Checkpoint Inhibitors. Front Immunol (2020) 11:783. 10.3389/fimmu.2020.00783 32508809 PMC7249937

[48] Theivanthiran B Evans KS DeVito NC Plebanek M Sturdivant M Wachsmuth LP. A Tumor-Intrinsic PD-L1/NLRP3 Inflammasome Signaling Pathway Drives Resistance to Anti-PD-1 Immunotherapy. J Clin Invest (2020). 10.1172/JCI133055 PMC7190922 32017708

[49] Murakami T Takigawa N Ninomiya T Ochi N Yasugi M Honda Y. Effect of AZD1480 in an Epidermal Growth Factor Receptor-Driven Lung Cancer Model. Lung Cancer (2014) 83:30–6. 10.1016/j.lungcan.2013.10.011 24238495

[50] Biffi G Oni TE Spielman B Hao Y Elyada E Park Y. IL1-Induced JAK/STAT Signaling Is Antagonized by TGFbeta to Shape CAF Heterogeneity in Pancreatic Ductal Adenocarcinoma. Cancer Discovery (2019) 9:282–301. 10.1158/2159-8290.CD-18-0710 30366930 PMC6368881

[51] Hamilton CE Craiglow BG. JAK Inhibitors for the Treatment of Pediatric Alopecia Areata. J Investig Dermatol Symp Proc (2020) 20:S31–6. 10.1016/j.jisp.2020.04.005 33099381

[52] Jerjen R Meah N Trinidade de Carvalho L Wall D Eisman S Sinclair R. Treatment of Alopecia Areata in Pre-Adolescent Children With Oral Tofacitinib: A Retrospective Study. Pediatr Dermatol (2020). 10.1111/pde.14422 33099833

[53] McInnes IB Byers NL Higgs RE Lee J Macias WL Na S. Comparison of Baricitinib, Upadacitinib, and Tofacitinib Mediated Regulation of Cytokine Signaling in Human Leukocyte Subpopulations. Arthritis Res Ther (2019) 21:183. 10.1186/s13075-019-1964-1 31375130 PMC6679539

[54] Severin F Frezzato F Visentin A Martini V Trimarco V Carraro S. In Chronic Lymphocytic Leukemia the JAK2/STAT3 Pathway Is Constitutively Activated and Its Inhibition Leads to CLL Cell Death Unaffected by the Protective Bone Marrow Microenvironment. Cancers (Basel) (2019) 11. 10.3390/cancers11121939 PMC6966457 31817171

[55] Fogelman D Cubillo A Garcia-Alfonso P Miron MLL Nemunaitis J Flora D. Randomized, Double-Blind, Phase Two Study of Ruxolitinib Plus Regorafenib in Patients With Relapsed/Refractory Metastatic Colorectal Cancer. Cancer Med (2018) 7:5382–93. 10.1002/cam4.1703 PMC6246927 30123970

[56] Verden A Dimbil M Kyle R Overstreet B Hoffman KB. Analysis of Spontaneous Postmarket Case Reports Submitted to the FDA Regarding Thromboembolic Adverse Events and JAK Inhibitors. Drug Saf (2018) 41:357–61. 10.1007/s40264-017-0622-2 29196988

[57] Rubbert-Roth A Enejosa J Pangan AL Haraoui B Rischmueller M Khan N. Trial of Upadacitinib or Abatacept in Rheumatoid Arthritis. N Engl J Med (2020) 383:1511–21. 10.1056/NEJMoa2008250 33053283

[58] Wohlford GF Van Tassell BW Billingsley HE Kadariya D Canada JM Carbone S. A Phase IB, Randomized, Double-Blinded, Dose Escalation, Single Center, Repeat-Dose Safety and Pharmacodynamics Study of the Oral NLRP3 Inhibitor Dapansutrile in Subjects With NYHA II-III Systolic Heart Failure. J Cardiovasc Pharmacol (2020). 10.1097/FJC.0000000000000931 PMC7774821 33235030

