# Supplementary figures and images for: Tumor NLRP3-Derived IL-1β Drives the IL-6/STAT3 Axis Resulting in Sustained MDSC-Mediated Immunosuppression
Source: Front Immunol. 2021 Aug 31;12:661323. doi: 10.3389/fimmu.2021.661323 (PMC8438323; doi:10.3389/fimmu.2021.661323)

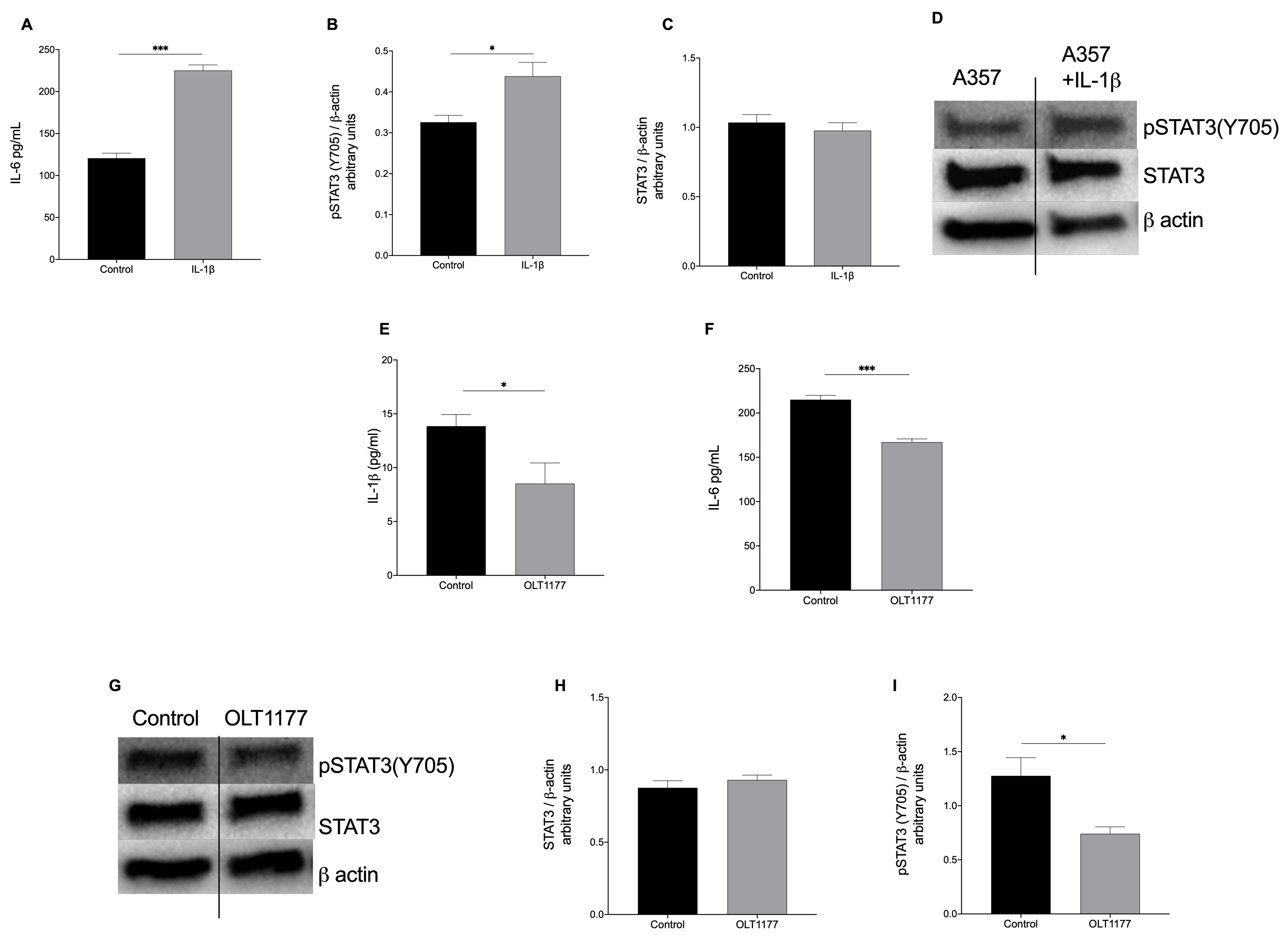

Supplement: Supplementary Figure 1 — (A) Mean ± SEM of IL-6 production from A357 cells stimulated with IL-1β after 24 hours (N=3). (B) Mean ± SEM of STAT3/β-actin ratio for 1205Lu cells shown in (A) (N=3). (C) Mean ± SEM of pSTAT3(Y705)/β-actin ratio for A357 cells shown in (a) (N=3). (D) Representative western blot images from (B, C). (E) Mean ± SEM of IL-1β production from unstimulated A357 cells treated with OLT1177 after 48 hours (N=3). (F) Mean ± SEM of IL-6 production from unstimulated A357 cells treated with OLT1177 after 48 hours (N=3). (G) Representative western blot images from (H) and (I). (H) Mean ± SEM of STAT3/β-actin ratio for A357 cells shown in (G) (N=3). (I) Mean ± SEM of pSTAT3(Y705)/β-actin ratio for A357 cells shown in (G) (N=3). *p < 0.05, ***p < 0.001. [file Image_1.tiff]

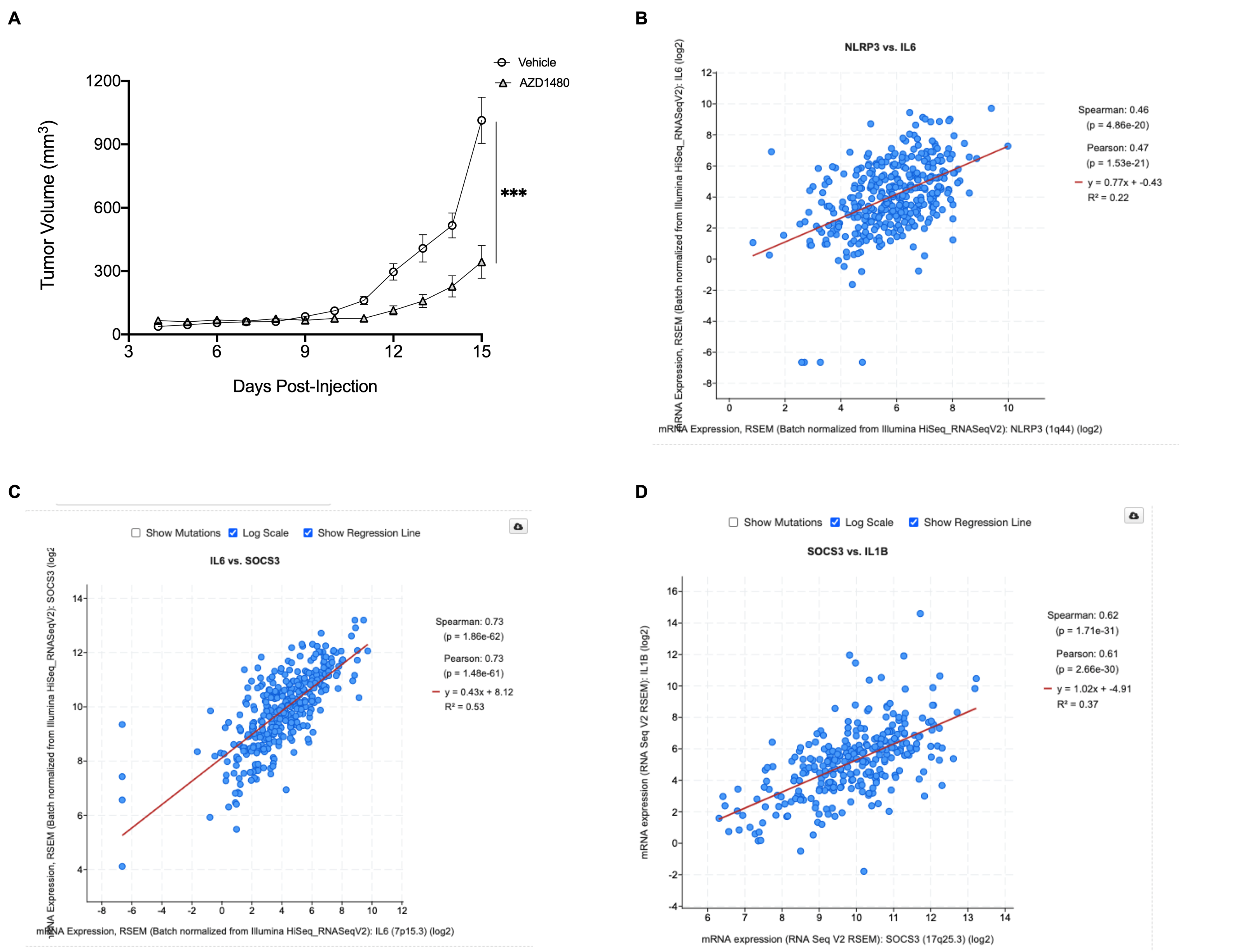

Supplement: Supplementary Figure 2 — (A)Tumor growth in mice treated with saline control (Vehicle) or AZD1480 (AZD1480) (N=8/group). (B) Correlation between NLRP3 and IL‐6 expression in human cutaneous melanoma (SKCM) TCGA datasets (N=363). (C) Correlation between of IL-6 and SOCS3 expression in human cutaneous melanoma (SKCM) TCGA datasets (N=363). (D) Correlation between of SOCS3 and IL-1β expression in human cutaneous melanoma (SKCM) TCGA datasets (N=363). [file Image_2.tiff]

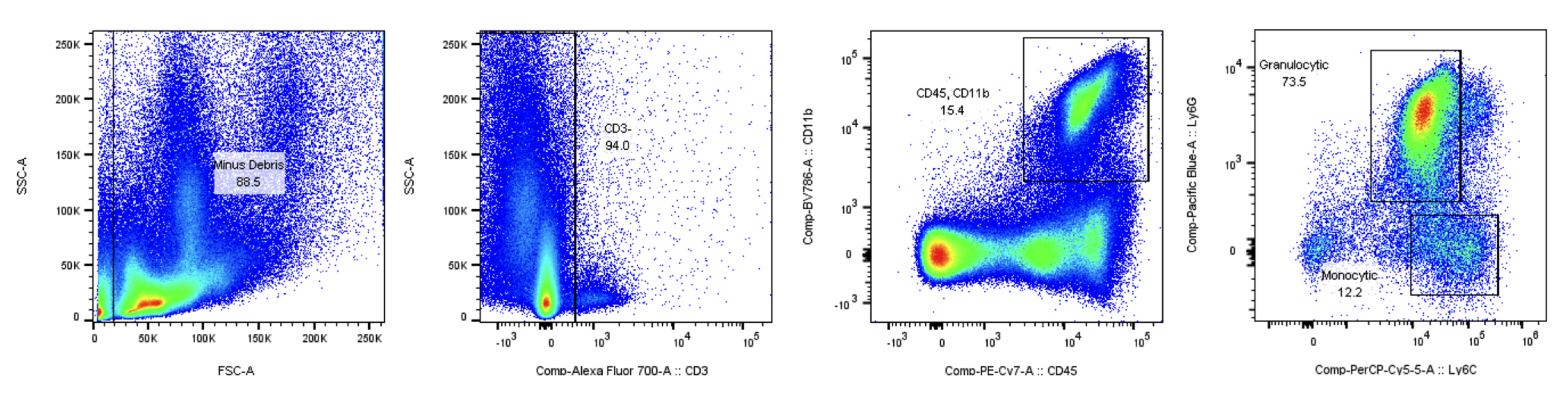

Supplement: Supplementary Figure 3 — FACs gating strategy depicting PMN-MDSCs populations that were isolated bone marrow of tumor-bearing mice. [file Image_3.tiff]

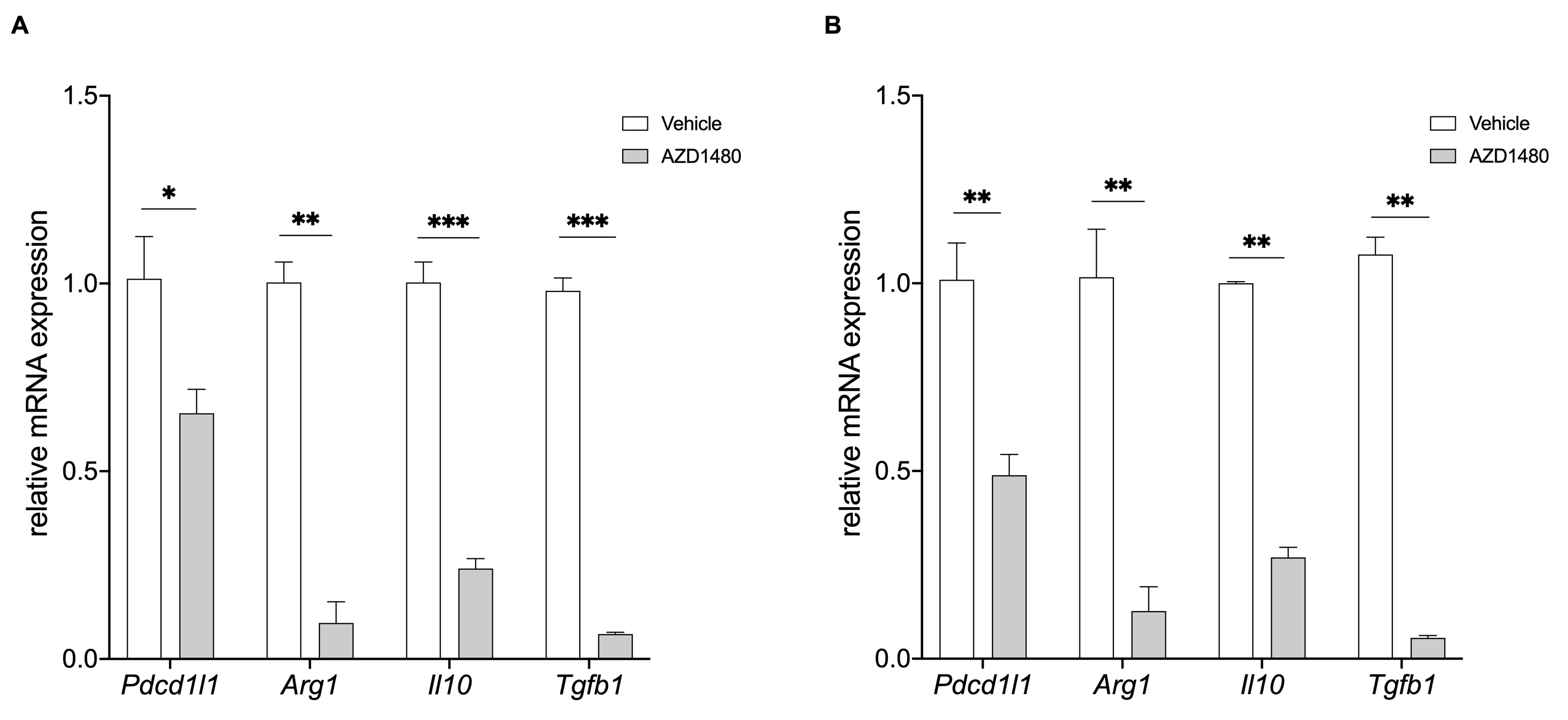

Supplement: Supplementary Figure 4 — Mice were implanted with B16F10 and treated with saline control (Vehicle) or AZD1480 (AZD1480), on day 15 PMN-MDSCs were isolated from bone marrow and spleen. (A) Mean ± SEM of relative mRNA expression of Pdcd1l1, Arg1, Il10 and Tgfb1 from PMN-MDSCs isolated from bone marrow of mice described above (N=2, pooled from 4 mice/group. 2 independent experiments). (B) Mean ± SEM of relative mRNA expression of Pdcd1l1, Arg1, Il10 and Tgfb1 from PMN-MDSCs isolated from bone marrow of mice described above (N=2, pooled from 4 mice/group. 2 independent experiments). *p < 0.05, **p < 0.001, ***p < 0.001. [file Image_4.tiff]
